# Supplementary material for: The Oncogenic Signaling Pathways in BRAF-Mutant Melanoma Cells Are Modulated by Naphthalene Diimide-Like G-Quadruplex Ligands
Source: Cells. 2019 Oct 18;8(10):1274. doi: 10.3390/cells8101274 (PMC6830342; doi:10.3390/cells8101274)
Supplement: Supplementary file 1 [file cells-08-01274-s001.pdf]

Supplementary Material

The Oncogenic Signaling Pathways in *BRAF*-Mutant Melanoma Cells are Modulated by Naphthalene Diimide-Like G-Quadruplex Ligands

Marta Recagni [1,†], Martina Tassinari [2,†], Filippo Doria [3], Graziella Cimino-Reale [1], Nadia Zaffaroni [1], Mauro Freccero [3], Marco Folini [1,*] and Sara N. Richter [2,*]

[1] Department of Applied Research and Technological Development, Fondazione IRCCS Istituto Nazionale dei Tumori di Milano, Via G.A. Amadeo 42, 20133 Milan, Italy; marta.recagni@istitutotumori.mi.it (M.R.); g.ciminoreale@virgilio.it (G.C.R.); nadia.zaffaroni@istitutotumori.mi.it (N.Z.)

[2] Department of Molecular Medicine, University of Padua, via A. Gabelli 63, 35121 Padua, Italy; martina.tassinari@unipd.it

[3] Department of Chemistry, University of Pavia, v. le Taramelli 10, 27100 Pavia, Italy; filippo.doria@unipv.it (F.D.); mauro.freccero@unipv.it (M.F.)

[*] Correspondence: marco.folini@istitutotumori.mi.it (M.F.); sara.richter@unipd.it (S.N.R.); Tel.: +39-022-390-5027 (M.F.); +39-049-827-2446 (S.N.R.)

[†] These authors contributed equally to this paper.

Synthetic Protocol

We synthesized the compounds **1–10, 13,** and **14** according to a published procedure [1–3], which was slightly modified to implement yields, as outlined in Scheme 1. We synthesized **11** and **12** using one-pot nucleophilic aromatic substitution and a Chichibabin-like reaction with **15-H**:**15-Br**/40:60 mixtures (Scheme 1). The raw mixture (0.5 mmol) was dissolved in 20 mL of dimethylformamide (DMF) in a round-bottom flask in the presence of 1.0 mmol of 4-chloro-*o*-phenylenediamine. The resulting solution was stirred at 80 °C, under argon. We monitored the reaction with analytical HPLC (see analytical method above). After 16 h, we quenched the resulting dark violet solution in water, extracting the suspension with three portions of $CHCl_3$ (50 mL each). The organic layers were collected, dried on Na_2SO_4, and evaporated with a vacuum. The crude product was dissolved with 0.1% trifluoroacetic acid (TFA) aqueous solution (0.5–1.2 mL) and purified via preparative HPLC using the preparative method. The purity of the collected fractions was evaluated by analytical HPLC, using the analytical method. See Supporting Information for the characterization of compounds **11** and **12** and their HPLC purity data. It has been reported that TFA anion might induce toxicity [4], therefore TFA was exchanged with a more biocompatible ion, such as chloride, by addition of 0.5 mL of 1 M HCl solution to each chromatographic portion. Solvent evaporation by vacuum presented the products as dihydrochloride (x2 HCl) salts.

Fluorescence Microscopy Analyses

Immunofluorescence analyses were carried out on cells grown on glass coverslips. Both untreated and treated cells were fixed in 4% formaldehyde/PBS for 15 min and incubated in a methanol/acetone solution for 15 min at room temperature. Cells were probed with a primary anti-G-quadruplex single chain antibody (BG4 300 ng/μL; dilution 1:20; produced as described in [5]) or a rabbit polyclonal anti-γ-H2AX antibody (ab11174; 1 mg/mL; dilution 1:500; Abcam, Cambridge, UK) for 1 h at room temperature (r.t.). To detect the G-quadruplex folding upon c-exNDI exposure, cells were probed with an anti-FLAG antibody (F1804, 1 mg/mL; dilution 1:800; Sigma-Aldrich (Milan, Italy)) for 1 h at RT. The AlexaFluor488 antibody (Life Technologies, A-11008) was used as a secondary antibody according to standard protocol. Nuclei were counterstained with 0.1 mg/mL of 4',6-diamidino-2-phenylindole (DAPI, Life Technologies, D1306).

Images were acquired using a Nikon Eclipse E600 microscope equipped with UV (nuclei) and FITC (γ-H2AX) filters by ACT-1 software (Nikon). Images were processed with Adobe Photoshop Image Reader 7.0. The uptake of c-exNDI 1 was detected by cell-imaging on the same samples using a TRITC filter. Pyridostatin (10 μM for 24 h; Tocris Bioscience, Bristol, UK) was used as a reference compound for G4 ligand-induced DNA damage.

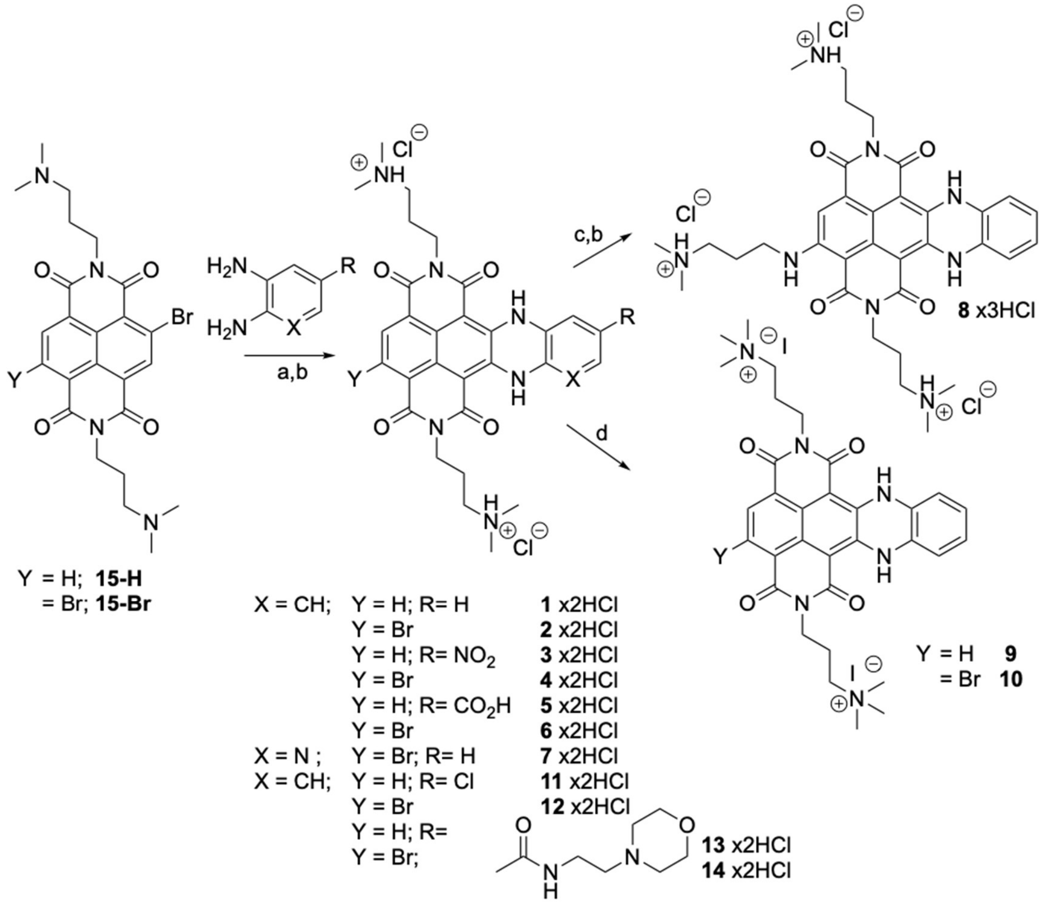

Scheme S1. Synthetic protocol for the preparation of core-extended naphthalene diimides (c-exNDIs). Reagents and conditions: (**a**) DMA, 80 °C, 16 h, under Ar; (**b**) HPLC purification and 1 M HCl addition/evaporation under vacuum; (**c**) N,N-dimethylpropylamine, MW, 140 °C, 4 min, closed vessel; (**d**) MeI, $CHCl_3$, r.t., 12 h.

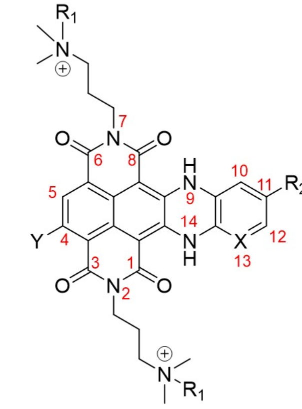

c-exNDI	X	Y	R_1	R_2
**1**	CH	H	H	H
**2**	CH	Br	H	H
**3**	CH	H	H	NO_2
**4**	CH	Br	H	NO_2
**5**	CH	H	H	COOH
**6**	CH	Br	H	COOH
**7**	N	Br	H	H
**8**	CH	$NH(CH_2)_3NMe_2$	H	H
**9**	CH	H	CH_3	H
**10**	CH	Br	CH_3	H
**11**	CH	H	H	Cl
**12**	CH	Br	H	Cl
**13**	CH	H	H	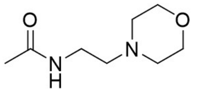
**14**	CH	Br	H	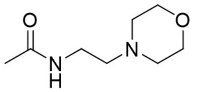

Figure S1. Structure and numbering of c-exNDIs. Compounds were prepared and tested as dihydrochloride (**1–7**, **11–14** × 2 HCl) or trihydrochloride (**8** × 3 HCl) salts. The quaternary ammoniums **9** and **10** were used as iodide salts.

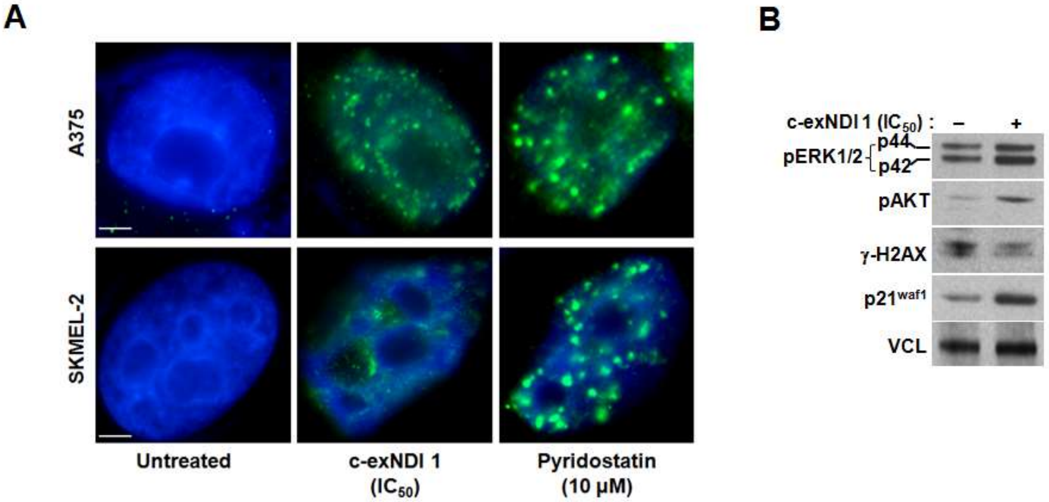

Figure S2. (**A**) Representative photomicrographs showing immunofluorescence analysis of DNA-damage induction (γ-H2AX foci, green fluorescence) in A375 and SKMEL-2 cells exposed for 48 h to an equitoxic amount of c-exNDI 1 (IC_{50}). Cells exposed to Pyridostatin (10 μM) were used as a positive control for G4 stabilization-mediated DNA-damage induction. Nuclei were counterstained with DAPI. Magnification: ×100; scale bar: 10 μm. (**B**) Representative Western immunoblotting showing the amounts of the indicated proteins in untreated (–) SKMEL-2 cells and upon 72 h of exposure (+) to **1** (IC_{50}). Vinculin (VCL) was used to ensure equal protein loading. Cropped images of selected proteins are shown.

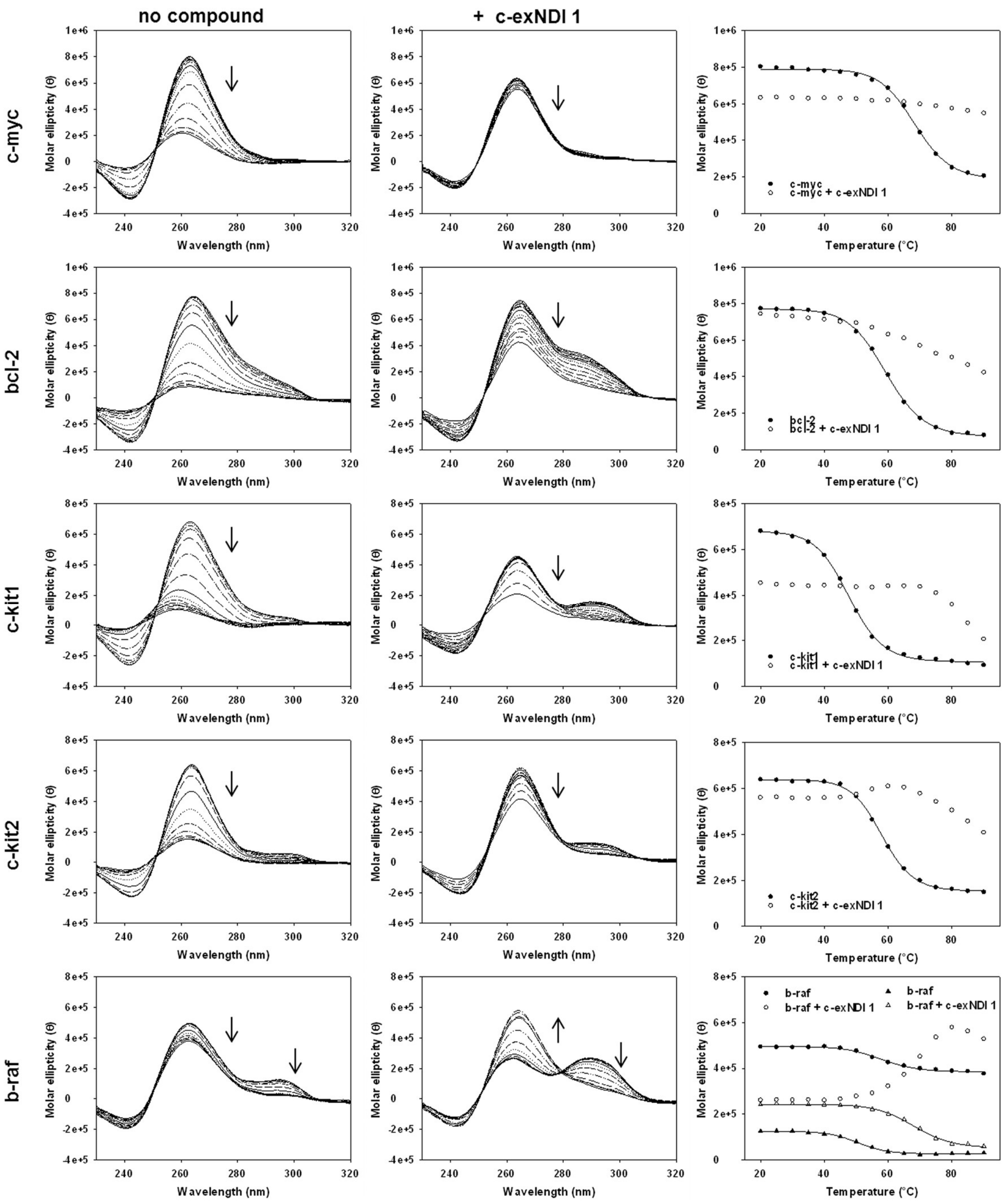

Figure S3. CD analysis of c-myc, bcl-2, c-kit1, c-kit 2 and b-raf G4 oligonucleotides (4 μM) in the absence (left panels) and presence (middle panels) of 4-fold excess of **1** (16 μM). The left and middle panels show CD spectra variation in function of the wavelength; arrows indicate the spectral change from 20 °C to 90 °C. The right panels show the molar ellipticity at the peak wavelengths as a function of the temperature, fitted with the vant' Hoff equation, where possible. Circles and triangles indicate the parallel (265 nm) and antiparallel (290 nm) G4 conformation contribution, respectively.

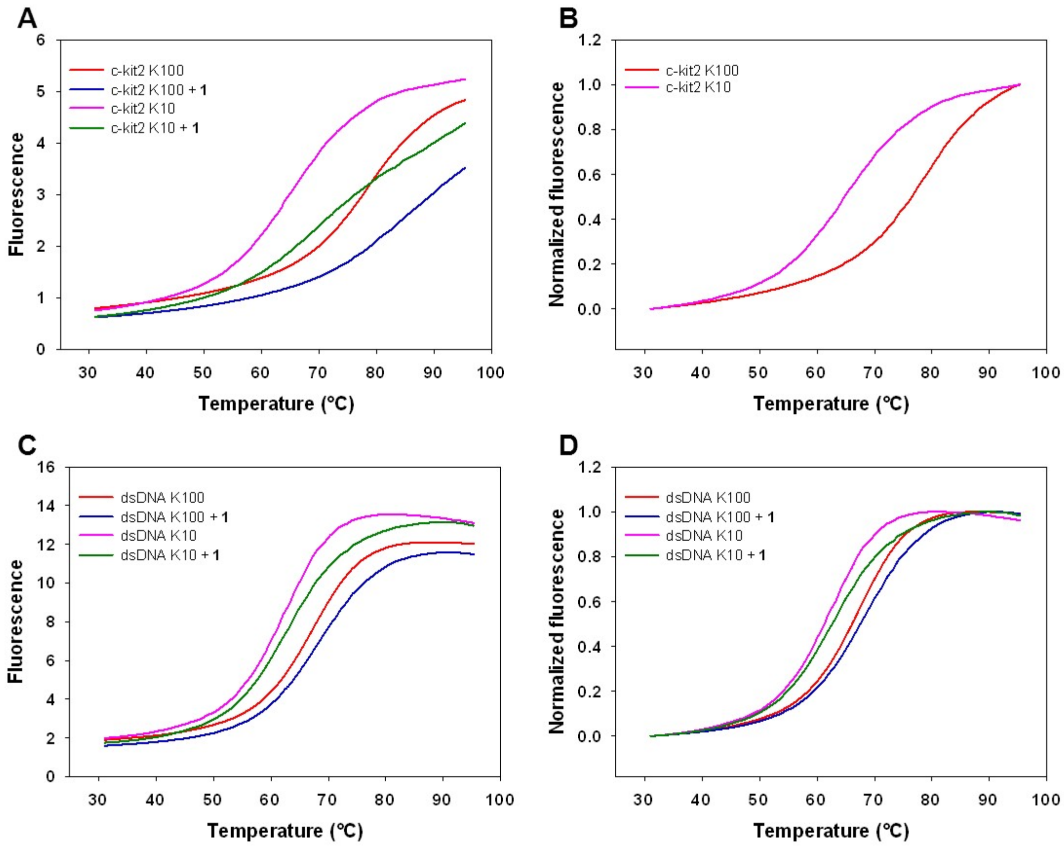

Figure S4. Representative FRET-melting curves of end-labeled c-kit2 (**A**-**B**) and dsDNA (**C**-**D**) 0.25 μM in the presence of **1** (0.25 μM) or the same amount of dimethyl sulfoxide (DMSO) at K^+ 100 mM or K^+ 10 mM. The right panels show the raw fluorescence, while the left panels show the normalized fluorescence data. Each condition was run in duplicate, and T_m was calculated from two independent experiments.

Table S1. Oligonucleotides used in this study.

Application	Name	Sequence (5′→ 3′)
**CD**	c-myc	TGGGGAGGGTGGGGAGGGTGGGGAAGG
	bcl-2	AGGGGCGGGCGCGGGAGGAAGGGGGCGGGAGCGGGGCTG
	b-raf	GGGCGGGGAGGGGGAAGGGA
	c-kit1	AGGGAGGGCGCTGGGAGGAGGGG
	c-kit2	CGGGCGGGCGCGAGGGAGGGG
**FRET**	c-kit 2	*FAM*[1] -CGGGCGGGCGCGAGGGAGGGG-*TAMRA*[2]
	dsDNA	*FAM*[1] -CTATAGCGCGCTATAG-*TAMRA*[2]
**Taq polymerase stop assay**	primer	GGCAAAAAGCAGCTGCTTATATGCAG
	c-myc	TTTTTGGGGAGGGTGGGGAGGGTGGGGAAGGTTTTTCTGCATATA AGCAGCTGCTTTTTGCC
	bcl-2	TTTTTAGGGGCGGGCGCGGGAGGAAGGGGGCGGGAGCGGGGCTG TTTTTCTGCATATAAGCAGCTGCTTTTTGCC
	b-raf	TTTTTGGGCGGGGAGGGGGAAGGGATTTTTCTGCATATAAGCAGC TGCTTTTTGCC
	c-kit1+2	TTTTTCGGGCGGGCGCGAGGGAGGGGAGGCGAGGAGGGGCGTGG CCGGCGCGCAGAGGGAGGGCGCTGGGAGGAGGGGTTTTTCTGCA TATAAGCAGCTGCTTTTTGCC
	non-G4 cnt	TTGTCGTTAAAGTCTGACTGCGAGCTCTCAGATCCTGCATATAAG CAGCTGCTTTTTGCC

[1] 6-carboxyfluorescein; [2] 6-carboxy-tetramethylrhodamine.

Table S2. Cytotoxic activity of **1** on primary skin fibroblasts and cell lines established from solid human tumors of different histological origins [1].

Cell line	Source [2]	IC_{50} (nM)	Doubling times (h) [3]
HDFa<br>Primary dermal fibroblasts from adult skin	Thermo Fisher Scientific, C0135C	>1000	227
A375<br>Malignant melanoma	ATCC® CRL-1619	9 ± 1	22
PC-3<br>Castration resistant prostate cancer cells	ATCC® CRL-1435™	12 ± 2	25
U-2 OS<br>Osteosarcoma cancer cells	ATCC® HTB-96™	14 ± 1	29.7
A-431<br>Epidermoid carcinoma	ATCC® CRL-1555™	14 ± 5	19.3
NCI-H460<br>Large cell lung cancer	ATCC® HTB-177™	24 ± 2	23
CaSki<br>Epidermoid carcinoma	ATCC® CRM-CRL-1550	40 ± 4	31.5
STO<br>Diffused malignant peritoneal mesothelioma	Perrone F, et al. [6]	59 ± 17	30
SiHa<br>Squamous cell carcinoma	ATCC® HTB-35	70 ± 4	35
SK-MEL-2<br>Malignant melanoma	ATCC® HTB-68	256 ± 30	32

[1] The cytotoxic activity of **1** was assessed using the MTS (3-(4,5-dimethylthiazol-2-yl)-5-(3-carboxymethoxyphenyl)-2-(4-sulfophenyl)-2H-tetrazolium) assay (CellTiter 96® AQueous One Solution Cell Proliferation Assay, Promega Italia, Milan, Italy). Briefly, 24 h after seeding, cells were treated with increasing concentrations (0.1–10,000 nM) of freshly diluted compounds and incubated at 37 °C and 5% CO_2 for 48 h. Cell viability was determined by incubating the cells for 4 h at 37 °C in the presence of 20 μL of MTS solution and by recording the absorbance, according to the manufacturer's instructions. The IC_{50} values (concentration of compound leading to 50% inhibition of cell viability) at each time point were calculated from the dose-response curves (percentage inhibition of cell viability with respect to cells exposed to solvent (DMSO) as a function of the Log_{10} concentrations of **1**) using GraphPad Prism 5.01 (GraphPad Software Inc., San Diego, CA, USA). Data are reported as mean values ± s.d. from at least three independent experiments. [2]ATCC: American Type Culture Collection (www.atcc.org). [3] Doubling times in hours were calculated according to the formula DoublingTime = duration ∗ log(2) / [log(Final Concentration) – log(Initial Concentration)].

Table S3. Analysis of gene expression levels in melanoma cells upon 48 h of exposure to **1** [1].

A375			SKMEL-2		
**Gene symbol**	**RQ**	**± s.d.**	**Gene symbol**	**RQ**	**± s.d.**
Down-regulated genes			*Down-regulated genes*		
AKT1	0.61	0.10	*BID*	0.47	0.00
APC	0.64	0.25	*FAS*	0.55	0.04
BCL2	0.65	0.11	*FGF2*	0.56	0.02
BCL2L1	0.46	0.02	*IGF1*	0.34	0.02
BRAF	0.55	0.02	*ITGAV*	0.58	0.01
CCND1	0.66	0.09	*ITGB3*	0.65	0.03
CDC42	0.57	0.00	*KIT*	0.67	0.05
CDK4	0.64	0.11	*MAP3K5*	0.65	0.03
CDKN1B	0.66	0.08	*NFKB2*	0.59	0.02
CYCS	0.44	0.05	*NFKBIA*	0.52	0.02
DVL1	0.59	0.06	*Up-regulated genes*		
EGFR	0.55	0.02	*CDH1*	1.80	0.06
ELK1	0.48	0.02	*CDKN1A*	1.93	0.07
ERBB2	0.65	0.03	*CDKN2B*	2.72	0.10
FN1	0.48	0.02	*CYCS*	1.56	0.06
FOS	0.52	0.02	*FADD*	1.59	0.06
FYN	0.50	0.06	*FASLG*	1.81	0.10
FZD1	0.66	0.00	*FN1*	1.52	0.00
GRB2	0.56	0.02	*FOS*	2.37	0.08
GSK3B	0.48	0.02	*ITGA2B*	2.41	0.10
IGF1R	0.46	0.05	*PTK2B*	2.26	0.11
ITGA2B	0.44	0.00	*Non-differently expressed genes*		
ITGB1	0.65	0.07	*ABL1*	1.04	0.04
ITGB3	0.49	0.05	*AKT1*	0.97	0.03
JUN	0.55	0.02	*AKT2*	0.84	0.09
LEF1	0.51	0.10	*APC*	1.23	0.00
MAP2K1	0.49	0.04	*BAX*	1.37	0.05
MAP3K5	0.33	0.07	*BCAR1*	1.40	0.08
MAPK1	0.30	0.04	*BCL2*	0.95	0.07
MAPK3	0.46	0.05	*BCL2L1*	1.08	0.01
MAPK8	0.44	0.03	*BCL2L11*	0.99	0.06
MAX	0.57	0.00	*BRAF*	0.85	0.03
MYC	0.65	0.07	*CASP8*	1.21	0.09
NFKB2	0.59	0.02	*CCND1*	1.47	0.05
NRAS	0.59	0.06	*CCND2*	1.02	0.03
PIK3CA	0.59	0.15	*CCND3*	0.88	0.06
PIK3R1	0.59	0.05	*CCNE1*	0.78	0.03
PTEN	0.58	0.04	*CDC42*	1.35	0.19
PTK2	0.58	0.04	*CDK2*	0.77	0.06
RAF1	0.53	0.08	*CDK4*	0.99	0.01

A375			SKMEL-2		
**Gene symbol**	**RQ**	**± s.d.**	**Gene symbol**	**RQ**	**± s.d.**
RB1	0.63	0.09	*CDKN1B*	0.76	0.05
RELA	0.62	0.04	*CDKN2A*	1.20	0.05
RHOA	0.49	0.02	*COL1A1*	1.49	0.04
SHC1	0.44	0.08	*CRK*	1.05	0.11

SMAD4	0.47	0.06	*CTNNB1*	1.16	0.08
SOS1	0.45	0.03	*DVL1*	0.76	0.01
SRC	0.55	0.02	*E2F1*	1.12	0.12
TCF3	0.62	0.09	*ELK1*	0.83	0.07
TGFB1	0.55	0.06	*ERBB2*	0.92	0.04
TGFBR1	0.58	0.04	*FYN*	1.16	0.02
TGFBR2	0.54	0.04	*FZD1*	0.85	0.03
TP53	0.50	0.03	*GRB2*	1.20	0.05
VEGFA	0.55	0.02	*GSK3B*	0.92	0.07
Up-regulated genes			*HRAS*	1.45	0.06
BAX	1.73	0.89	*IGF1R*	1.06	0.05
BCL2L11	2.10	0.17	*ITGB1*	1.21	0.13
CDKN1A	1.70	0.07	*JUN*	0.98	0.04
Non-differently expressed genes			*KDR*	0.94	0.01
ABL1	0.69	0.14	*KRAS*	0.89	0.06
AKT2	0.67	0.30	*LEF1*	1.17	0.06
BCAR1	1.08	0.15	*MAP2K1*	1.19	0.04
BID	0.82	0.11	*MAPK1*	1.00	0.00
CASP8	0.96	0.13	*MAPK14*	0.91	0.04
CCND2	1.02	0.04	*MAPK3*	0.76	0.00
CCND3	0.81	0.00	*MAPK8*	1.08	0.01
CCNE1	0.89	0.10	*MAX*	1.20	0.05
CDK2	0.71	0.09	*MDM2*	0.78	0.03
CDKN2A	0.83	0.03	*MYC*	0.74	0.03
COL1A1	0.81	0.06	*NFKB1*	0.85	0.03
CRK	0.91	0.07	*NRAS*	1.12	0.04
CTNNB1	0.73	0.08	*PIK3CA*	0.98	0.08
E2F1	0.71	0.10	*PIK3R1*	0.79	0.03
FADD	1.05	0.11	*PTEN*	0.93	0.06
FAS	1.12	0.04	*PTK2*	1.00	0.07
FGF2	0.85	0.03	*RAC1*	1.12	0.04
HRAS	0.68	0.03	*RAF1*	0.87	0.06
ITGAV	0.72	0.03	*RB1*	0.79	0.03
KDR	0.87	0.06	*RELA*	0.91	0.04
KRAS	1.15	0.08	*RHOA*	0.82	0.01
MAPK14	0.98	0.08	*SHC1*	1.36	0.05
MDM2	0.81	0.06	*SMAD4*	1.05	0.04
NFKB1	0.76	0.09	*SOS1*	0.87	0.00
NFKBIA	0.85	0.03	*SPP1*	0.90	0.03
	**A375**			**SKMEL-2**	
**Gene symbol**	**RQ**	**± s.d.**	**Gene symbol**	**RQ**	**± s.d.**
PTK2B	1.21	0.74	*SRC*	0.84	0.03
RAC1	0.79	0.03	*TCF3*	1.04	0.09
SPP1	0.92	0.10	*TGFB1*	0.90	0.03
Undetected genes			*TGFBR1*	1.12	0.04
CASP9	-	-	*TGFBR2*	1.05	0.04
CDH1	-	-	*TP53*	1.01	0.01
CDKN2B	-	-	*VEGFA*	1.34	0.10
FASLG	-	-	***Undetected genes***		
HGF	-	-	*CASP9*	-	-
IGF1	-	-	*EGFR*	-	-

KIT	-	-	*HGF*	-	-
WNT1	-	-	*WNT1*	-	-

[1] Gene expression data are reported as Relative Quantity (mean RQ values ± s.d.). The cut-off for differently expressed genes in treated vs. untreated cells was set at fold-change |1.5| (RQ < 0.67 or RQ > 1.5). Undetected genes had no signal after 40 cycles of PCR amplification.

Table S4. Over-representation analysis showing the most relevant enriched pathways found in the list of differently expressed genes (with a fold-change >|2.0|) in treated vs. untreated melanoma cells [1].

**A375**		
geneSet	***Description***	***p Value***
R-HSA-6802952	Signaling by BRAF and RAF fusions	0.004397796
R-HSA-6802957	Oncogenic MAPK signaling	0.004397796
R-HSA-74749	Signal attenuation	0.005990102
R-HSA-6802949	Signaling by RAS mutants	0.015058934
R-HSA-5674135	MAP2K and MAPK activation	0.015058934
R-HSA-6802946	Signaling by moderate-kinase activity BRAF mutants	0.015058934
R-HSA-6802948	Signaling by high-kinase activity BRAF mutants	0.015058934
R-HSA-6802955	Paradoxical activation of RAF signaling by kinase-inactive BRAF	0.015058934
R-HSA-422475	Axon guidance	0.015275749
R-HSA-448424	Interleukin-17 signaling	0.016669598
**SKMEL-2**		
geneSet	***Description***	***p Value***
R-HSA-114608	Platelet degranulation	0.064656041
R-HSA-76005	Response to elevated platelet cytosolic Ca^{2+}	0.064656041
R-HSA-2559582	Senescence-associated secretory phenotype (SASP)	0.174019732
R-HSA-2559580	Oxidative stress-induced senescence	0.199304842
R-HSA-9018519	Estrogen-dependent gene expression	0.291819116
R-HSA-212436	Generic transcription pathway	0.31782553
R-HSA-73857	RNA polymerase II transcription	0.31782553
R-HSA-74160	Gene expression (transcription)	0.31782553
R-HSA-3700989	Transcriptional regulation by TP53	0.333572253
R-HSA-450341	Activation of the AP-1 family of transcription factors	0.340659177

[1] WEB-based GEne SeT AnaLysis Toolkit (WebGestalt, http://webgestalt.org).

HPLC purity data

Compound **11**

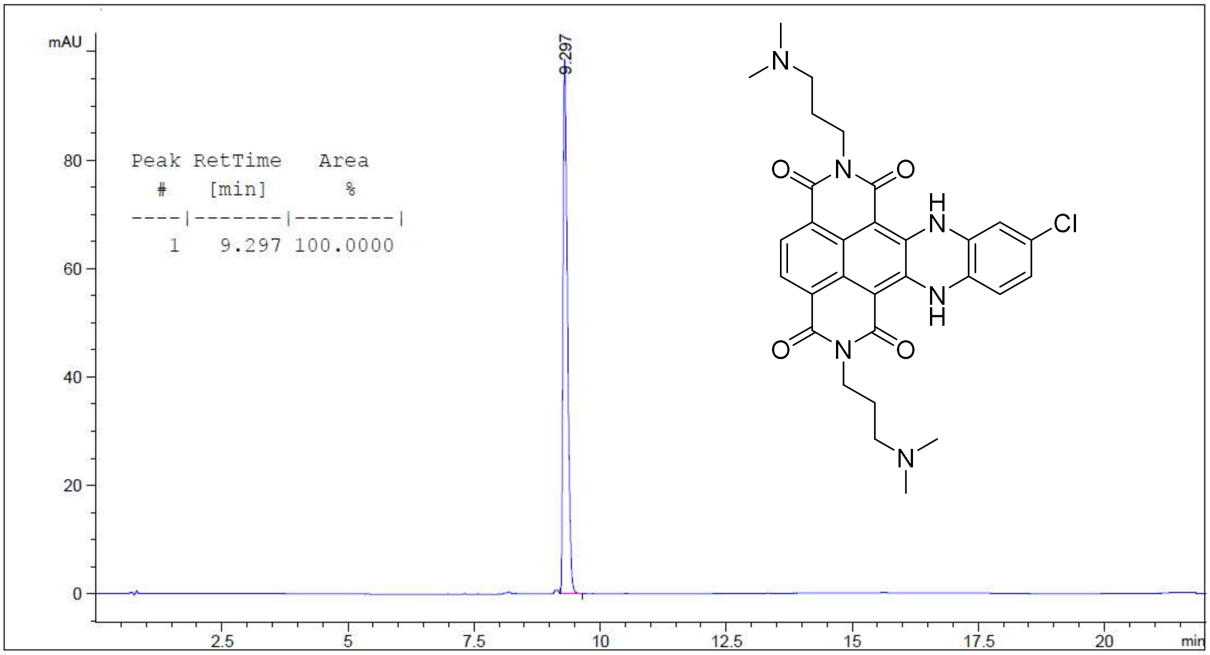

Compound **12**

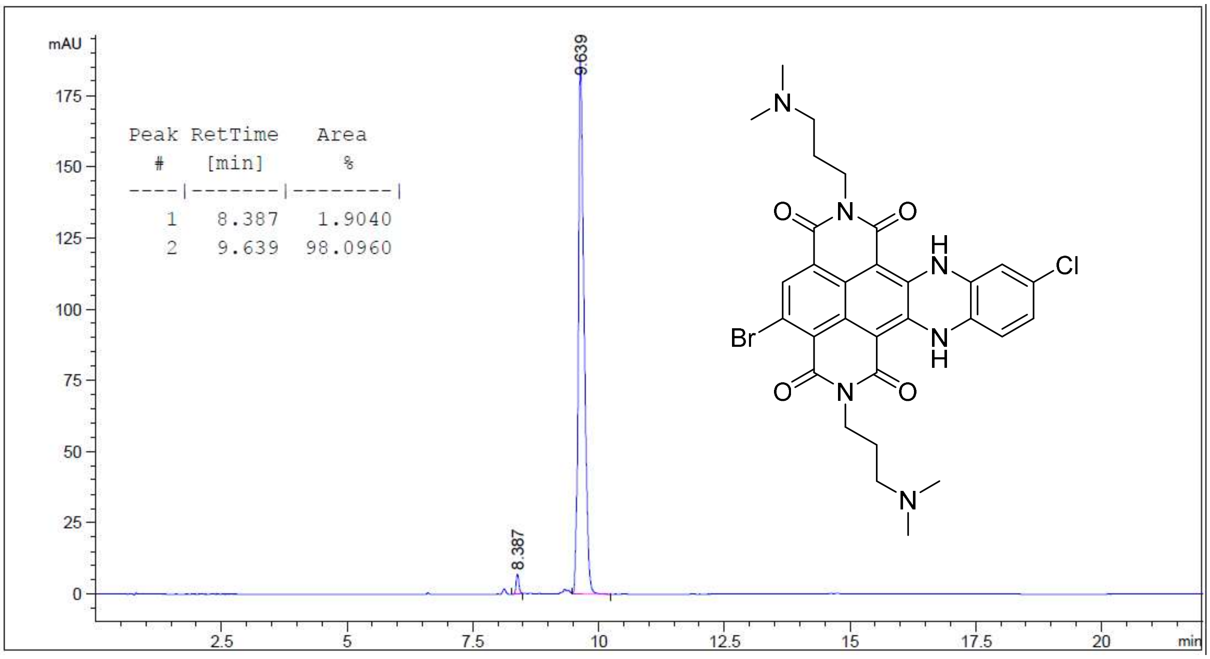
mAU
175
150
125
100
75
50
25
0
2.5
5
7.5
10
12.5
15
17.5
20
min
9.639
8.387
Peak RetTime Area
[min] %
1 8.387 1.9040
2 9.639 98.0960
N
O
N
O
H
N
Cl
Br
N
H
O
N
O
N

NMR characterization

Compound **11**

^{1}H NMR-300 MHz CD$_3$OD

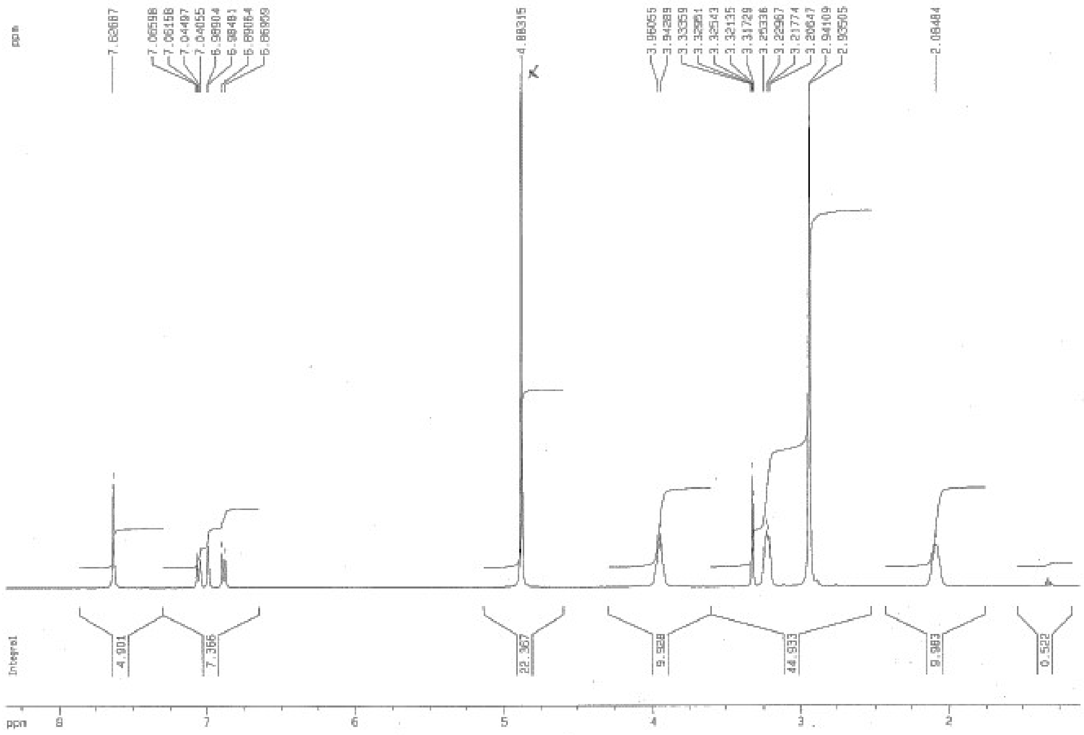

^{13}C NMR-300 MHz CD_3OD

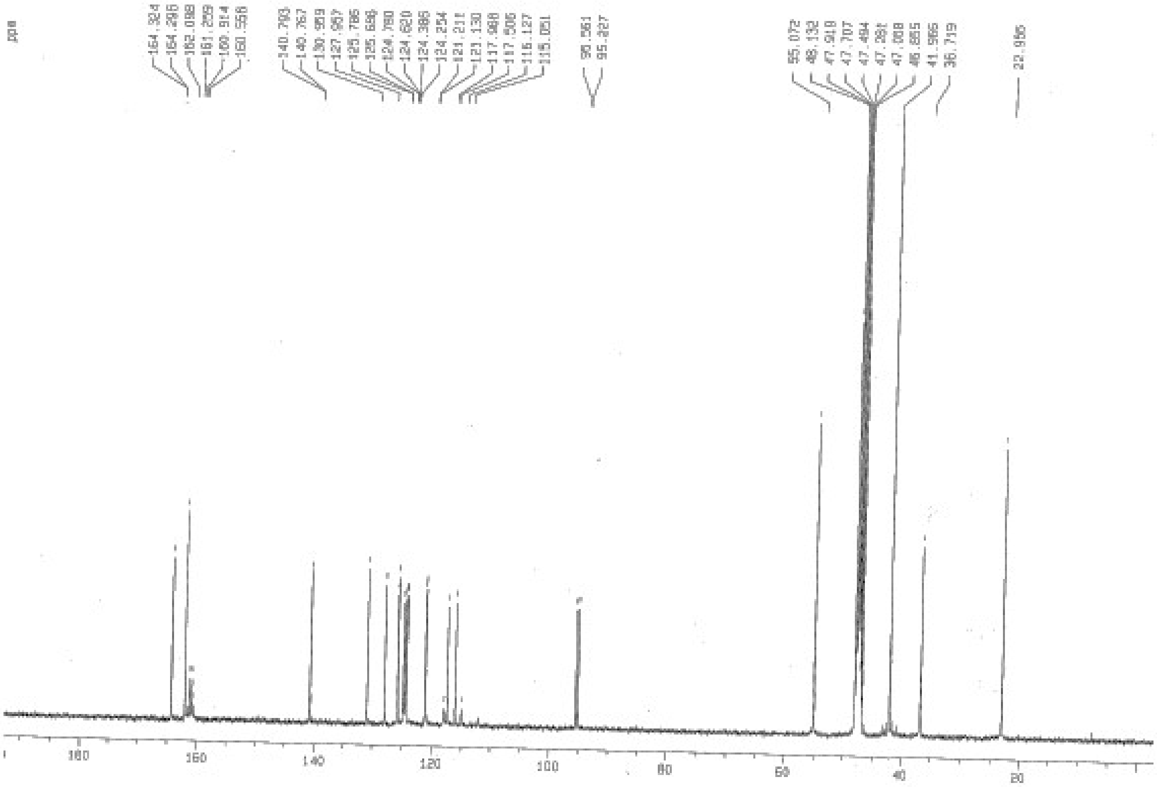

Compound **12**

^{1}H NMR-300 MHz CD_3OD

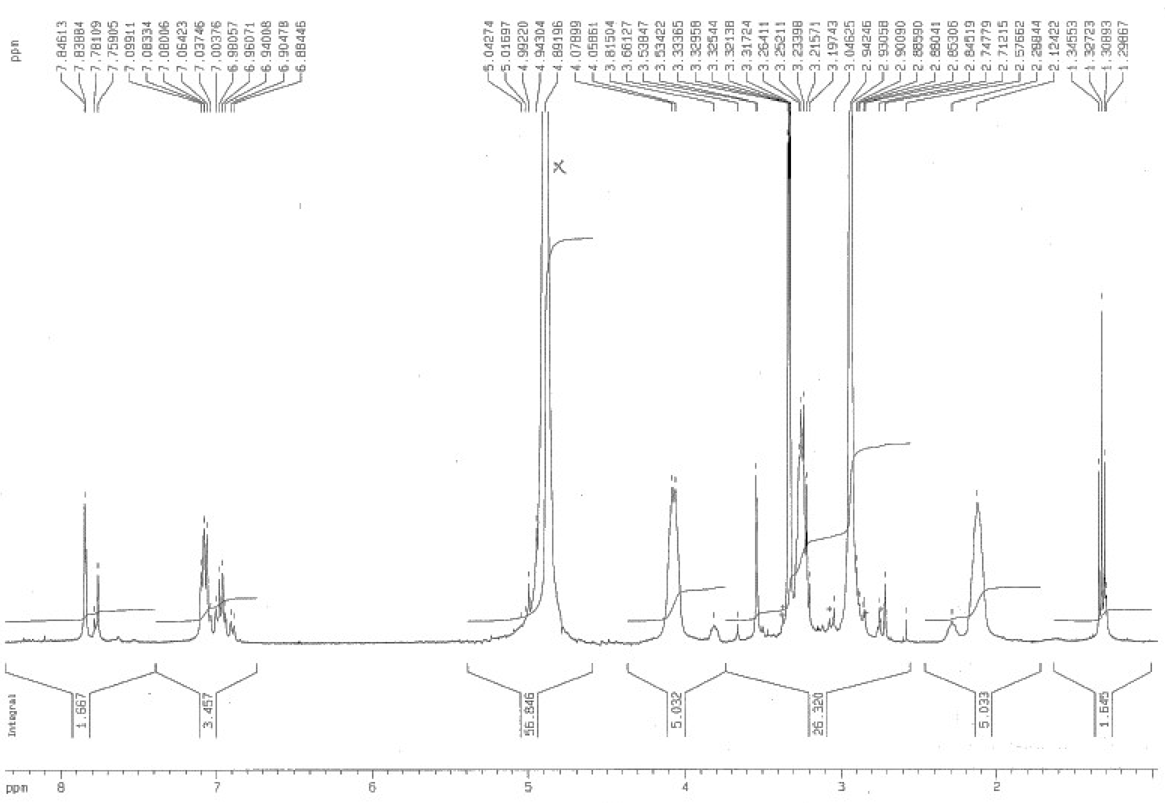

^{13}C NMR-300 MHz CD$_3$OD

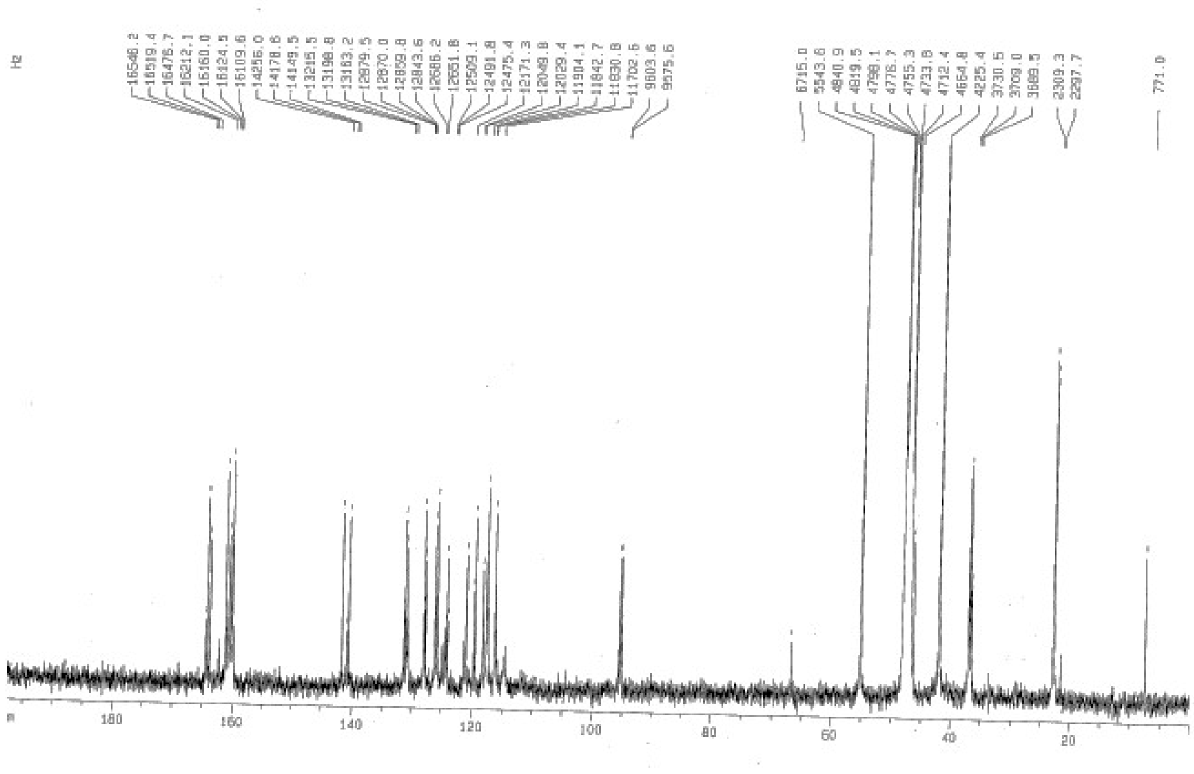

Characterization of c-exNDIs 11 and 12

Compound **11**·3HCl: Blue solid (yield 38%; m.p. dec. > 200 °C). ^{1}H-NMR (400 MHz, CD_3OD): 7.63 (s, 2 H), 7.05 (dd, 1 H, *J* = 8 Hz; 1.8 Hz), 6.98 (d, 1 H, *J* = 1.8 Hz), 6.88 (d, 1 H, *J* = 1.8 Hz), 3.95 (bs, 4 H), 3.23 (m, 4 H), 2.93 (s, 12 H); 2.08 (bs, 4 H). ^{13}C-NMR (100 MHz, CD_3OD): 164.3;164.2; 140.8; 131.0; 128.0; 125.8; 125.7; 124.8; 124.6; 124.4; 124.2; 121.2; 121.1; 117.5; 116.1; 95.6; 95.3; 55.1; 42.0; 36.7; 23.0.

Compound **12**·3HCl: Blue solid (yield 22%; m.p. dec. > 200 °C). ^{1}H-NMR (400 MHz, CD_3OD): 7.85 (s, 1 H), 7.08 (m, 2 H), 6.99 (m, 1 H), 4.07 (m, 4 H), 3.23 (m, 4 H), 2.94 (s, 12 H); 2.12 (bs, 4 H). ^{13}C-NMR (100 MHz, CD_3OD): 165.4; 165.1; 162.1;161.1; 142.5; 141.5; 132.1; 131.9; 128.8; 128.7; 126.8; 126.5; 125.1; 121.7; 120.3; 119.0; 118.3; 117.0; 96.0; 95.8; 55.4; 42.2; 37.1; 37.3; 23.1; 23.0.

Supplementary References

1. Perrone, R.; Doria, F.; Butovskaya, E.; Frasson, I.; Botti, S.; Scalabrin, M.; Lago, S.; Grande, V.; Nadai, M.; Freccero, M.; et al. Synthesis, Binding and Antiviral Properties of Potent Core-Extended Naphthalene Diimides Targeting the HIV-1 Long Terminal Repeat Promoter G-Quadruplexes. *J. Med. Chem.* **2015**, *58*, 9639–52.
2. Zuffo, M.; Guédin, A.; Leriche, E.-D.; Doria, F.; Pirota, V.; Gabelica, V.; Mergny, J.-L.; Freccero, M. More is not always better: finding the right trade-off between affinity and selectivity of a G-quadruplex ligand. *Nucleic Acids Res.* **2018**, *46*, e115.
3. Zuffo, M.; Doria, F.; Botti, S.; Bergamaschi, G.; Freccero, M. G-quadruplex fluorescence sensing by core-extended naphthalene diimides. *Biochim. Biophys. acta. Gen. Subj.* **2017**, *1861*, 1303–1311.
4. Cornish, J.; Callon, K.E.; Lin, C.Q.-X.; Xiao, C.L.; Mulvey, T.B.; Cooper, G.J.S.; Reid, I.R. Trifluoroacetate, a contaminant in purified proteins, inhibits proliferation of osteoblasts and chondrocytes. *Am. J. Physiol. Metab.* **1999**, *277*, E779–E783.
5. Gilbert-Girard, S., Gravel, A., Artusi, S., Richter, S.N., Wallaschek, N., Kaufer, B.B., Flamand, L. Stabilization of Telomere G-Quadruplexes Interferes with Human Herpesvirus 6A Chromosomal Integration. *J Virol*. **2017**, *91*, pii: e00402-17.
6. Perrone, F.; Jocollè, G.; Pennati, M.; Deraco, M.; Baratti, D.; Brich, S.; Orsenigo, M.; Tarantino, E.; De Marco, C.; Bertan, C.; et al. Receptor tyrosine kinase and downstream signalling analysis in diffuse malignant peritoneal mesothelioma. *Eur. J. Cancer* **2010**, *46*, 2837–48.
